# The Songbird as a Percussionist: Syntactic Rules for Non-Vocal Sound and Song Production in Java Sparrows

**DOI:** 10.1371/journal.pone.0124876

**Published:** 2015-05-20

**Authors:** Masayo Soma, Chihiro Mori

**Affiliations:** 1 Department of Biology, Faculty of Science, Hokkaido University, Kita 10, Nishi 8, Kita-ku, Sapporo, Hokkaido 060–0810, Japan; 2 Biosystems Science Course, Graduate School of Life Science, Hokkaido University, Kita 10, Nishi 8, Kita-ku, Sapporo, Hokkaido 060–0810, Japan; Texas Christian University, UNITED STATES

## Abstract

Music and dance are two remarkable human characteristics that are closely related. Communication through integrated vocal and motional signals is also common in the courtship displays of birds. The contribution of songbird studies to our understanding of vocal learning has already shed some light on the cognitive underpinnings of musical ability. Moreover, recent pioneering research has begun to show how animals can synchronize their behaviors with external stimuli, like metronome beats. However, few studies have applied such perspectives to unraveling how animals can integrate multimodal communicative signals that have natural functions. Additionally, studies have rarely asked how well these behaviors are learned. With this in mind, here we cast a spotlight on an unusual animal behavior: non-vocal sound production associated with singing in the Java sparrow (*Lonchura oryzivora*), a songbird. We show that male Java sparrows coordinate their bill-click sounds with the syntax of their song-note sequences, similar to percussionists. Analysis showed that they produced clicks frequently toward the beginning of songs and before/after specific song notes. We also show that bill-clicking patterns are similar between social fathers and their sons, suggesting that these behaviors might be learned from models or linked to learning-based vocalizations. Individuals untutored by conspecifics also exhibited stereotypical bill-clicking patterns in relation to song-note sequence, indicating that while the production of bill clicking itself is intrinsic, its syncopation appears to develop with songs. This paints an intriguing picture in which non-vocal sounds are integrated with vocal courtship signals in a songbird, a model that we expect will contribute to the further understanding of multimodal communication.

## Introduction

Investigating how animals coordinate their movements with the sounds they produce holds the key to understanding the evolution of communicative and musical cognitive abilities. Research into the coordination of movement with sound is topical, and shows that some species other than humans can also spontaneously synchronize their movements with musical rhythms to which they are exposed [1–5]. However, only a few studies have addressed the question of how animals can temporally coordinate naturally produced multimodal communicative signals (e.g., vocalizations and movement).

Although many bird species engage in ritualized courtship displays in which visual and auditory signals are integrated in ways that emphasize their signals, quantitative assessment of the degree to which these signals are coordinated is rare. A study investigating the courtship displays of male Barbary doves (*Streptopelia risoria*) reported that bowing and calling were synchronized on a fine time scale [6]. Similarly fine temporal synchronization of spreading wings and song was reported to occur in the courtship display of male brown-headed cowbirds (*Molothrus ater*) [7]. Further, male superb lyrebirds (*Menura novaehollandiae*) coordinate their dance repertoires with different song types [8], while in the zebra finch (*Taeniopygia guttata*) the relationship between dance movement and song elements exists but is not obligate [9]. To show both mechanistic and functional aspects of such coordination in avian courtship displays, considering the degree to which the combinations of vocal and gestural elements are conserved among individuals within a species is worthwhile. If a particular motion is always associated with a specific vocalization or with the timing of sequential vocalizations, that could suggest intrinsic constraints (e.g., [7]), though such stereotyped motions might not add rich information to the signal. Additionally, despite extensive data regarding the process and mechanisms of social song learning, how individuals develop or learn to integrate cross-modal signals remains a mystery [10–12]. Interestingly, Williams [9] reported that father/son pairs of the zebra finch exhibited similar patterns of coordinated dance and song element sequences, suggesting that the choreography is socially learned (though the sample size was very small and genetic inheritance could not be excluded as an alternative explanation).

In this study, we investigate multimodal coordination in communication using a novel animal model in which songbirds integrate and coordinate non-vocal sound production with learned songs. While the majority of birds rely on vocal communication, some species, mostly non-vocal learners, are known for using mechanical sounds for communication. These include the feather sounds of some manakins [13–15], the wing-whistle sounds of the crested pigeon (*Ocyphaps lophotes*) [16], woodpecker drumming [17], and the drumming display of the ruffed grouse (*Bonasa umbellus*) [18].

Here, we focus on non-vocal sound production in male Java sparrows (*Lonchura oryzivora*; order: Passeriformes, family: Estrildidae), which produce bill-click sounds along with their songs during courtship displays (directed singing) [19, 20], as well as when they sing to themselves when alone (undirected singing). Moreover, we have never observed bill clicking in Java sparrows that were not singing, and to the best of our knowledge such a phenomenon has never been reported in the literature. We assume that the bill click sounds are an important component of their courtship signals. The clicking is associated with a very slight grinding movement of the bill (S1 and S2 Movies), but exactly how it is produced is still unknown. Such intriguing behavior offers an ideal opportunity to investigate how non-vocal sounds are synchronized with songs and how the overall pattern is learned.

## Materials and Methods

### Ethics Statement

This study was conducted with approval from the Institutional Animal Care and Use Committee of the National University Corporation at Hokkaido University (No. 11–0028) in accordance with Hokkaido University Regulations of Animal Experimentation. During the study, stress was minimized and all birds were cared for and treated appropriately in accordance with the Guidelines for Proper Conduct of Animal Experiments from the Science Council of Japan and the Guidelines for Ethological Studies from the Japan Ethological Society. After the study, birds were used either for other experiments or for breeding purposes.

### Experimental procedures

We investigated individual differences in bill-click frequency, the coordination between song notes and bill clicks, and bill-click learnability by analyzing archived recordings of undirected songs made by male Java sparrows. Our dataset included recordings from 30 domesticated adult males, of which 22 were known to be related, including nine fathers and their genetic sons (n = 10) and foster sons (n = 3). The other males (n = 8) were reared in experimentally controlled social environments in which bill-click sounds were absent—seven in isolation and untutored after 30 days old (untutored birds, see below for details), and one with a pair of Bengalese finches (*Lonchura striata* var. domestica) that do not possess this behavior (heterospecific-fostered bird). Male Java sparrows sing one song type that they learn from their social fathers [21]. The songs are characterized by their note-type repertoire and the ordering of stereotyped notes [22, 23], and often involve bill clicks. To best compare song learning and bill clicks, birds that failed to copy the full note repertoire of their fathers (n = 3) were not used as subjects in this study.

Birds were housed with their family (i.e., a breeding pair and siblings) in breeding cages (45 × 45 × 45 cm^3^), maintained in a controlled environment (25 ± 3°C, 30%–60% humidity, 12L:12D photoperiod), and provided with finch seed mixture, foxtail millet coated with egg yolk, rice, water, shell grit, and green vegetables ad libitum.

The majority of the 30 test animals were laboratory bred (though a few were untutored individuals obtained from a pet store; see below). Non-fostered individuals were kept in the breeding cage until their songs had crystallized at around 180 days of age. Fostered individuals were placed in the cage of their foster parents from just prior to hatching until song crystallization. After each bird was temporarily placed in a cage within a sound attenuated room, we recorded its song using a digital audio recorder (Marantz PMD 661) with a sampling rate of 44.1 kHz and 16-bit resolution. The untutored birds (n = 7) obtained from the pet store (Koizumi, Sapporo) were between 20 and 30 days old. They were socially isolated from conspecifics and individually hand raised in a sound attenuated box until they could feed themselves. Their singing behavior was monitored and recorded continuously throughout the day using Sound Analysis Pro v1.40 [24] with a sampling rate of 44.1 kHz and 16-bit resolution. As these untutored birds did not sing often, we used recordings over a span of multiple days to obtain around 20 song samples per individual. The age of these untutored individuals was estimated in relation to the median day of their sampled songs.

**Fig 1:**
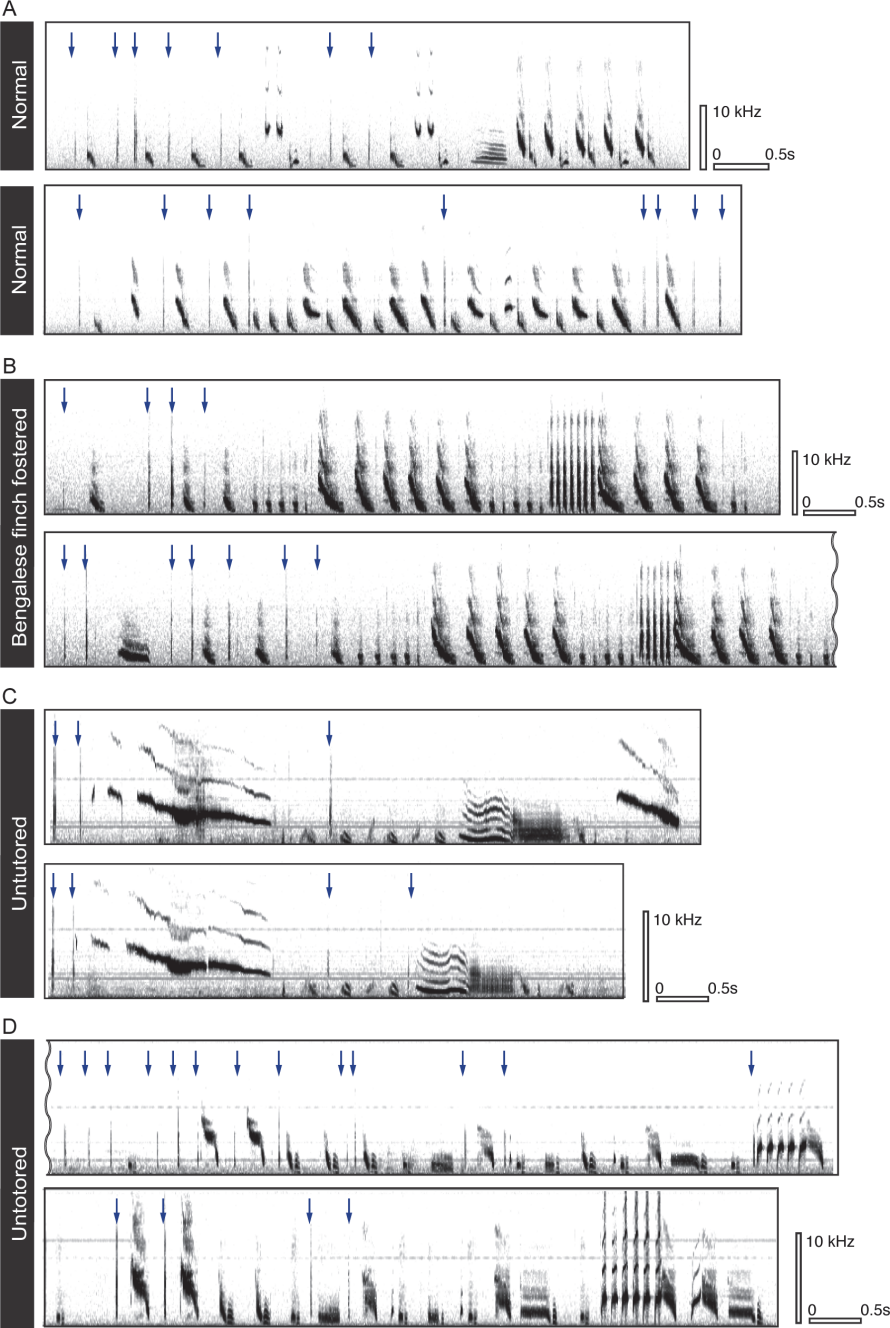
Sonograms of the songs of male Java sparrows with bill clicks. Songs of two conspecific-tutored normally reared birds (A), songs of an individual reared by a Bengalese finch pair (B), and songs from two birds untutored after day 30 of age (C, D). Bill clicks are indicated with arrows.

On average, 23.8 songs were sampled from each subject (n = 30). We visualized song data, identified bill clicks, and categorized song notes using Raven Pro 1.4 sound analysis software [25]. Bill clicks were distinguished from song notes by their acoustic structures visible in sonograms: bill clicks are shorter than most song notes (approx. 0.01–0.02 s), and characterized by harsh broad-band energy that is different from that exhibited by song notes that contain frequency modulations or tonal/harmonic structures (Fig 1). Bill clicks were also distinguishable from the noise made by moving birds on the basis of their short duration, but generally recorded songs did not involve such sounds as Java sparrow males stand still and do not move around during undirected singing. In rare cases where the timing of bill clicks and song notes overlapped, they were discarded from analyses. When clicks were overlapped by harmonic-structured notes, they were clearer in sonograms, but otherwise clicks could be masked by song notes and less detectable even if they existed. To exclude such note-specific detectability, we decided not to consider them.

We used logistic regression to investigate whether age and rearing environments (i.e., reared by conspecifics or not) affect the probability that an individual will sing with bill clicks. We then analyzed how bill clicking was integrated with song-note sequences in the birds that used clicks in > 60% of their songs, by using the odds of presence/absence of clicks before/after specific note types. We used logistic regression to test whether bill clicking was preceded or followed by specific note types (referred to as ‘pre’ and ‘post’ note types in the results), or whether it was produced at random positions in the note sequence. Finally, we assessed the learnability of bill-clicking patterns. We used a generalized linear mixed model (GLMM) with binomial distribution to test the strength of the association between the bill-click probability displayed by a father for each note-to-note transition type and his son’s click probability for the corresponding transitions. To account for the effect of song learning, we also included the song similarity between fathers and sons (assessed as the proportion of note-to-note transition repertoires that sons learned from their fathers) as an explanatory variable. We included song family and bird identity as random effects.

## Results

### Individual variations in bill clicking

**Fig 2:**
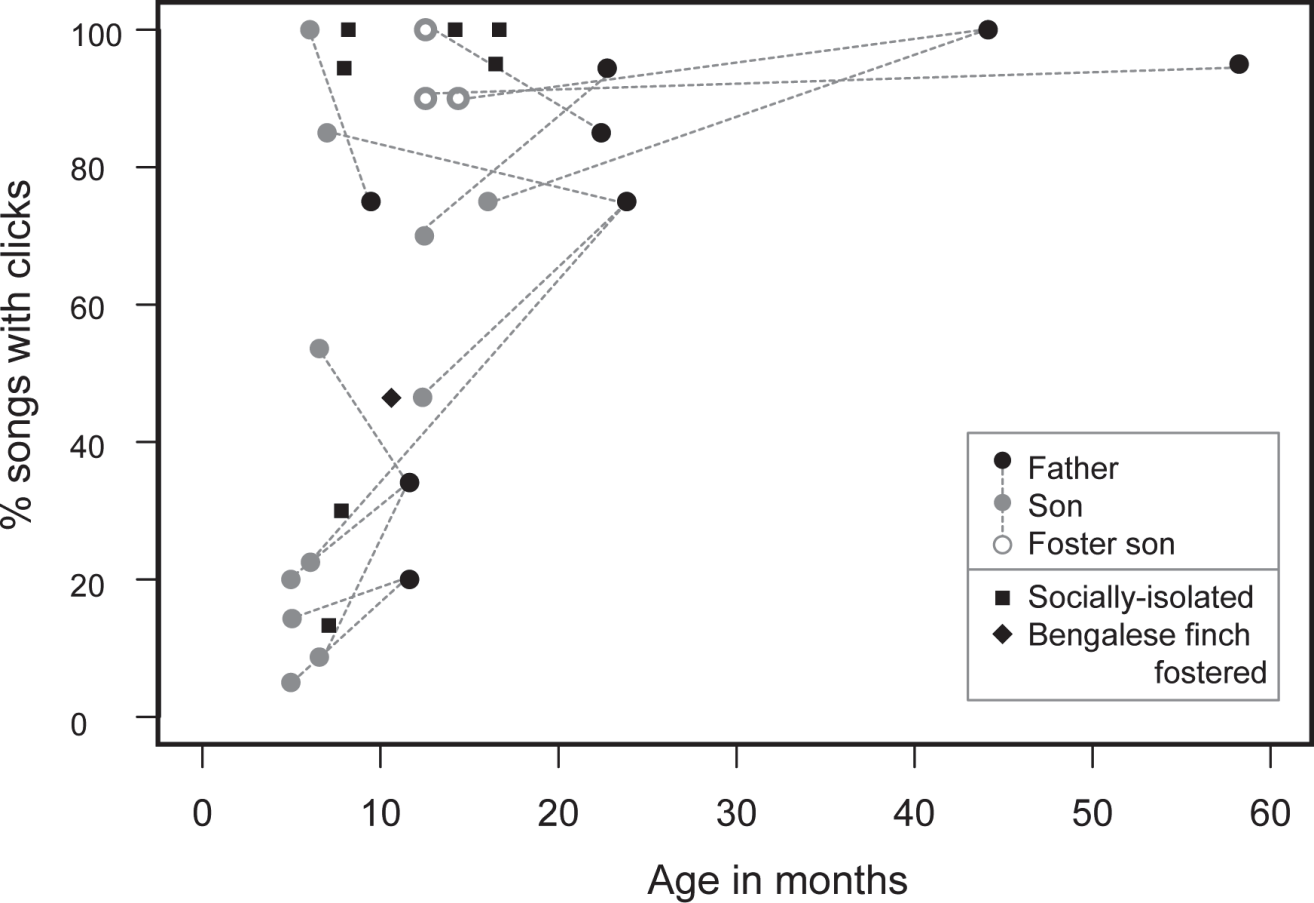
Age related increase in the rate of bill clicks. The proportion of songs with bill clicks in Java sparrow males from conspecific families (n = 22), untutored (n = 7), and heterospecific-fostered (n = 1) is shown as a function of the age of the individual. Fathers and sons are connected with dashed lines. The age effect was still significant when we reanalyzed the data excluding two outliers (the eldest two with frequent bill clicks).

We confirmed that all male birds, including those never exposed to conspecific tutors (untutored and heterospecific-fostered birds) produced songs with bill clicks (Figs 1 and 2, S1 Audio). Overall, 59.0% (422/715) of the recorded songs included bill clicks, though proportions varied extensively across individuals (5%–100%). Our analysis revealed that age, but not rearing environment, accounted for some of this variation amongst individuals (GLM, age: *p* < 0.05, rearing environment: *p* = 0.52; Fig 2). Older birds produced bill clicks during almost every song bout, while younger adults did not, suggesting that bill clicks continue to be added after song crystallization at around the age of 6 months. Moreover, fathers and sons showed a similar rate of clicks (Spearman’s rank correlation: r = 0.713, *p* < 0.03), which suggests that the observed individual variation is not random, but rather depends on social learning or genetic inheritance.

### Syntactic rules of bill clicking

**Fig 3:**
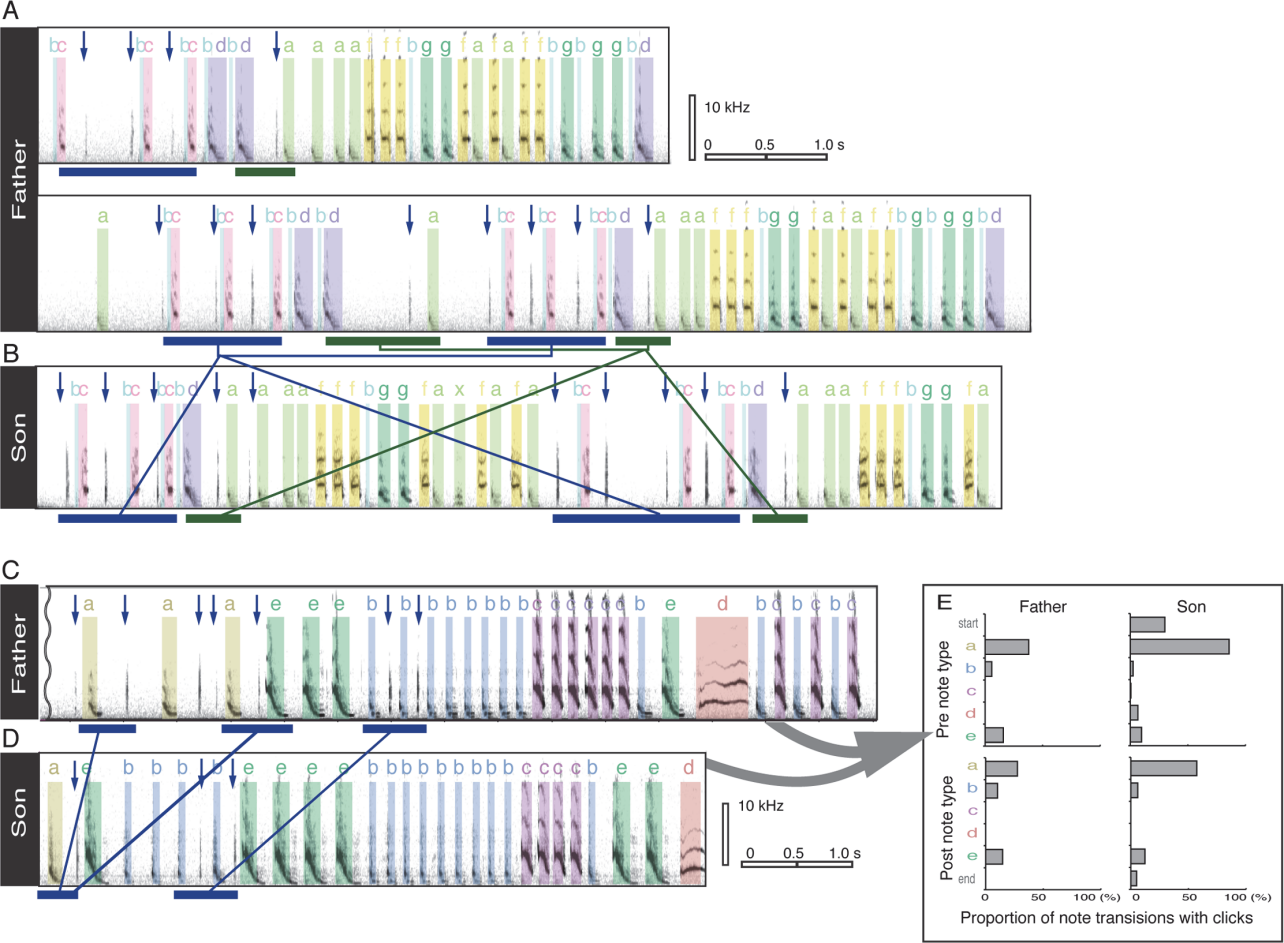
Sonograms of songs with bill clicks for two father-son pairs (A–D). Different song notes are individually colored, clicks are indicated with arrows, and connected blue and green bars show similar patterns of note-click coordination between fathers and sons. For illustrative purposes, we have chosen to show songs with a greater number of bill clicks. For the last father-son pair, the proportion of bill clicks that was preceded or followed by each note type is shown in four histograms (E), revealing that click-note coordination was well preserved along with song learning. This information for other father-son pairs is provided in S2 Table.

Eighteen birds showed frequent bill clicks (clicks in > 60% of songs) and their data were further analyzed to establish whether bill clicking was integrated within the syntactic rules of the song-note sequences in a consistent manner. We found that bill clicks were well coordinated with song-note sequences in both conspecific-reared and untutored individuals. Specifically, every male Java sparrow produced bill clicks at a significantly higher rate before and/or after specific note types (S1 Table, Figs 1 and 3), indicating that the birds’ bill clicks are associated with specific syllables. As shown in Fig 1 however, clicks were emitted more frequently at the beginning of songs, allowing the possibility that the association between note types and clicks was mediated by note order (i.e., both particular note types and bill clicks were produced earlier in song phrases). To resolve this, we took note order into account when analyzing the pattern of bill clicking in the latter halves of note sequences (latter half was from the middle to last notes, defined by the number of notes involved in each song). Eight out of 18 birds (44%) showed frequent bill clicks in the latter halves of songs (clicks in >50% of songs) and their behaviors were analyzed. We found that every bird produced bill clicks at a significantly higher rate before and/or after specific note types within the second halves of songs (S1 Table), but that note order effect was weak and not statistically significant in most birds (S1 Table). We also found that the number of clicks per transition and the temporal position within the note-to-note interval were variable even within individuals (Figs 1 and 3).

### Learnability of the bill-click-song coordination

To determine whether associations between clicks and song notes are learned, we compared bill-click rates in the note transitions shared between social fathers and sons, taking into account the effect of song learning. Sons learned 79.2 ± 8.2% (range: 68.4%–90.0%) of their fathers’ transition repertoires, and this individual variation in song learning had a significant effect on the bill-click rate (GLMM with binomial error distribution, effect of song learning: β = 6.53, SE = 2.07, z = 3.16, *p* < 0.002), which indicates that ‘good’ song learners produced more bill clicks.

Moreover, we found that across seven father-son pairs (fathers: n = 6, genetic sons: n = 4; foster sons: n = 3), the bill-click rate of sons was significantly higher at note transitions during which their fathers also tended to produce clicks (GLMM with binomial error distribution, effect of fathers’ click rates: β = 4.06, SE = 0.24, z = 17.2, *p* < 0.001; Fig 3, S2 Table, S2 Audio). This similarity in song-associated bill clicking patterns between fathers and sons was also statistically significant when we analyzed foster and genetic sons separately (GLMM with binomial error distribution, genetic sons: β = 8.05, SE = 0.46, z = 17.7, *p* < 0.001; foster sons: β = 2.24, SE = 0.27, z = 8.24, *p* < 0.001). Additionally, we found that sons tended to produce multiple clicks at the same note transitions during which their fathers produced multiple clicks (GLMM with Poisson error distribution, effect of fathers’ multi-click rates: β = 1.96, SE = 0.64, Z = 3.04, *p* < 0.003). A supplemental analysis revealed that song learning (assessed as the proportion of transitions learned from fathers) and click learning (the proportion of transition-specific click occurrences learned from fathers) were not significantly correlated (Spearman’s rank correlation: r = 0.28, *p* = 0.54).

## Discussion

This study reports the novel finding that the bill-click sound produced by male Java sparrows is coordinated and potentially learned with its song. We showed that male sparrows that were not tutored by conspecifics also sang with bill clicks, indicating that the bill-click behavior itself is intrinsic and not learned. However, while the act of bill clicking seems intrinsic, we also demonstrated that individual bill-clicking patterns are closely integrated with specific song-note sequences (Figs 1 and 3), and these associations are shared between social fathers and their sons. This important finding supports the idea that bill-click production in relation to note sequence is culturally transmitted, but whether clicking patterns themselves were learned or whether they were simply linked to learning-based vocalizations is still debatable.

There remains much room for discussion about whether and how species that learn their vocalizations synchronize their movements with sounds, but at least it is known that beak movement plays a part in vocalizations. Williams [9] reported that beak opening and closing was associated with song-note sequence in the zebra finch. In songbirds, variable beak opening functions as a filter and is responsible for acoustics features of song syllables [26, 27]. Considering that clicks are presumably produced from nearly closed beaks (see S2 Movie), beak postures associated with specific acoustic features of song notes might be a factor that explains the production of bill clicks immediately before or after specific note types in the Java sparrow. However, the association between song notes and clicks was not perfect and allowed for some variability. This suggests that mechanisms other than fixed vocalization-associated beak movements could lie behind the improvised percussionist-like performance of the Java sparrow. This view is also supported by the fact that additional bill clicks were inserted into the songs after song learning period (Fig 2). Further insights could be obtained by investigating the ontogeny of bill-clicking behavior in conjunction with detailed investigation into the physiological and neural mechanisms driving this behavior.

The question of why Java sparrows use non-vocal sound communication in addition to singing remains a puzzle. Interestingly, the bill is sexually dimorphic and exhibiting swelling in both sexes when they are reproductively active [20, 28]. Preliminary observations have revealed that female Java sparrows also produced bill clicks (S1 and S2 Movies). Hence, song could be a directional courting signal from male to female Java sparrows, while bill clicks might function as interactive signals between the sexes (S1 and S2 Movies). Mirroring behaviors (copying gestures or vocal duets) are an affiliative form of communication that can contribute to the formation and maintenance of social bonds in a range of animal taxa [29–32]. Such behaviors may be selected for because these cognitive abilities enable coordination between oneself and others. Although many studies tend to look into exaggerated multimodal sexual signals that have evolved as male traits under intense sexual selection pressure (as in lek breeding system) [8, 13–15], insight into mutual signaling between sexes could help us understand why complex ways of communication have also evolved in species like the Java sparrow that have monogamous breeding systems [33].

## Supporting Information

S1 Movie Typical courtship display shown by a pair of Java sparrows. A female is on the cage floor while a male perches above. Both birds produce bill clicks while the male sings his song. They copulate after the female completes her copulation-solicitation display.
(MOV)

S2 Movie Female bill-clicking behavior. Slow speed version of S1 Movie shows the slight bill movement associated with click-sound production.
(MOV)

S1 Audio Songs with bill clicks in Bengalese finch-fostered and untutored birds. Six songs that correspond to Fig 2B, 2C and 2D.
(AIF)

S2 Audio Songs with bill clicks in normally reared birds. Five songs that correspond to Fig 3.
(WAV)

S1 Table The effects of preceding and following note types on click probability in note sequences for the songs of each bird. (PDF)

S2 Table Comparisons of bill-click syntax between fathers and sons. (PDF)
