# Supplementary material for: The Songbird as a Percussionist: Syntactic Rules for Non-Vocal Sound and Song Production in Java Sparrows
Source: PLoS One. 2015 May 20;10(5):e0124876. doi: 10.1371/journal.pone.0124876 (PMC4438869; doi:10.1371/journal.pone.0124876)
Supplement: S1 Table — (PDF) [file pone.0124876.s005.pdf]

Table S1. The effects of preceding and following note types on click probability in note sequences of the songs of each subject. **(A)** To test if the male Java sparrow produced bill-clicks before or after particular note types, we fitted the data with logistic regression models, in which the dependent variable was whether or not each transition involved clicks and the explanatory variables were preceding and following note types of the transition (referred to as pre and post note types). The statistical significance of the effects of pre- and post-note types were derived from a log-likelihood ratio test comparing the full model (pre and post) with two models that dropped one of the terms (either pre or post) from the full model. **(B)** We also applied the above analyses to the latter half of note sequences of songs, given the possible confounding effect of note orders (i.e., bill clicks and particular notes tended to be produced in earlier part of songs). In these models, the dependent variable was whether or not each transition involved clicks and the explanatory variables were preceding and following note types of the transition, and note orders. Due to the fact that some birds produced bill clicks only in earlier parts of songs, subject birds were limited compared with (A). **(C)** Comparisons of bill-click syntax between the first and last halves are shown for the songs analyzed in (B)

(A) Whole note sequences of songs

Song family	ID		Note repertoire	Analysed songs	Pre note type $\chi2$	Pre note type p	Post note type $\chi2$	Post note type p
A	js0002	Father	5	n = 20	**74.40**	**< 0.0001**	**21.93**	**< 0.0001**
A	js0048	Foster son	5	n = 20	**22.29**	**< 0.0001**	3.96	0.161
B	js0003	Father	7	n = 20	**68.04**	**< 0.0001**	**25.88**	**< 0.0001**
B	js0042	Son	7	n = 20	**25.26**	**< 0.0001**	3.47	0.435
B	js0044	Foster son	7	n = 20	**9.03**	**0.012**	**11.26**	**0.002**
D	js0016	Father	6	n =16	**17.65**	**< 0.0001**	**33.09**	**< 0.0001**
D	js0069	Son	6	n = 20	**20.72**	**< 0.0001**	**78.92**	**< 0.0001**
E	js0023	Father	6	n = 20	5.13	0.114	**18.14**	**< 0.0001**
E	js0051	Foster son	6	n = 18	**40.66**	**< 0.0001**	**34.13**	**< 0.0001**
G	js0037	Father	5	n = 18	**25.40**	**< 0.0001**	**14.32**	**< 0.0001**
G	js0052	Son	5	n = 20	**21.60**	**< 0.0001**	**11.55**	**< 0.0004**
B2	js0041	Father	6	n =16	**27.67**	**< 0.0001**	**7.44**	**0.021**
B2	js0087	Son	6	n = 20	**47.33**	**< 0.0001**	**14.60**	**< 0.0001**
Untutored	prpl045		11	n = 20	**24.32**	**< 0.0001**	**21.56**	**< 0.0001**
Untutored	Ltgr142		7	n = 18	**9.00**	**0.012**	**26.60**	**< 0.0001**
Untutored	Ltgr073		5	n = 20	0.19	0.984	**70.68**	**< 0.0001**
Untutored	Ltgr031		10	n = 20	**44.92**	**< 0.0001**	**51.34**	**< 0.0001**
Untutored	Ltgr047		8	n = 20	**23.77**	**< 0.0001**	**11.90**	**< 0.003**

(B) Latter half note sequences of songs

Song family	ID		Pre note type $\chi2$	Pre note type p	Post note type $\chi2$	Post note type p	Order $\chi2$	Order p
A	js0002	Father	**39.88**	**< 0.0001**	**9.75**	**0.0006**	1.23	0.134
B	js0044	Foster son	**10.45**	**0.002**	**8.46**	**0.018**	0.33	0.417
E	js0051	Foster son	**8.73**	**0.002**	**13.29**	**< 0.0001**	0.22	0.510
B2	js0087	Son	**18.50**	**< 0.0001**	**9.78**	**0.003**	1.75	0.062
Untutored	prpl045		**13.45**	**0.003**	5.52	0.440	0.81	0.203
Untutored	Ltgr142		**23.92**	**< 0.0001**	**18.33**	**< 0.0001**	1.61	0.073
Untutored	Ltgr073		0.29	0.900	**26.33**	**< 0.0001**	**4.39**	**0.036**
Untutored	Ltgr031		**23.59**	**< 0.0001**	**9.38**	**0.016**	**7.29**	**0.007**

(C) Comparisons of bill-click syntax between the first and last halves of the songs analyzed in (B)

JS0002	First half			Second half		
Pre note type		n	click		n	click
start	50%	20	10	-	-	-
a	46%	83	38	47%	100	47
b	0%	28	0	2%	44	1
c	8%	105	8	1%	87	1
d	0%	4	0	0%	11	0
e	93%	14	13	-	0	0
Post note type						
a	47%	92	43	32%	91	29
b	3%	33	1	3%	39	1
c	15%	110	17	20%	82	16
d	0%	5	0	0%	10	0
e	57%	14	8	-	0	0
end	-	-	-	15%	20	3

JS0044	First half			Second half		
Pre note type		n	click		n	click
start	10%	20	2	-	-	-
a	17%	111	19	9%	35	3
b	6%	109	6	2%	81	2
c	10%	82	8	0%	23	0
d	0%	6	0	17%	29	5
e	7%	15	1	18%	50	9
f	7%	15	1	3%	92	3
g	17%	12	2	19%	48	9
post note type						
a	19%	115	22	16%	31	5
b	8%	116	9	4%	74	3
c	6%	86	5	0%	19	0
d	0%	10	0	0%	25	0
e	7%	15	1	8%	50	4
f	7%	15	1	13%	92	12
g	8%	13	1	15%	47	7
end	-	-	-	0%	20	0

JS0051	First half			Second half		
Pre note type		n	click		n	click
start	0%	18	0	-	-	-
a	42%	74	31	-	0	0
b	22%	67	15	7%	55	4
c	1%	184	1	4%	190	8
d	0%	6	0	0%	38	0
e	-	0	0	0%	21	0
f	-	0	0	5%	37	2
post note type						
a	42%	74	31	-	0	0
b	12%	67	8	4%	55	2
c	4%	198	7	2%	176	4
d	0%	8	0	6%	36	2
e	50%	2	1	26%	19	5
f	-	0	0	3%	37	1
end	-	-	-	0%	18	0

JS0087	First half			Second half		
Pre note type		n	click		n	click
start	65%	20	13	-	-	-
a	25%	75	19	16%	62	10
b	0%	77	0	2%	58	1
c	52%	52	27	56%	9	5
d	60%	20	12	23%	13	3
f	0%	37	0	0%	86	0
g	0%	6	0	0%	60	0
Post note type						
a	35%	82	29	11%	55	6
b	51%	81	41	13%	54	7
c	2%	53	1	0%	8	0
d	0%	20	0	0%	13	0
f	0%	42	0	0%	81	0
g	0%	9	0	2%	57	1
end	-	-	-	25%	20	5

prpl045	First half			Second half		
Pre note type		n	click		n	click
start	15%	20	3	-	-	-
a	0%	7	0	0%	2	0
b	1%	77	1	0%	4	0
c	18%	74	13	0%	3	0
d	0%	4	0	0%	13	0
e	0%	11	0	4%	54	2
f	0%	35	0	2%	47	1
g	0%	4	0	0%	12	0
h	60%	5	3	53%	36	19
i	50%	12	6	10%	51	5
j	54%	24	13	38%	24	9
k	-	0	0	0%	18	0
Post note type						
a	29%	7	2	0%	2	0
b	5%	77	4	0%	4	0
c	11%	75	8	0%	2	0
d	0%	4	0	0%	13	0
e	0%	15	0	2%	50	1
f	0%	44	0	3%	38	1
g	0%	5	0	0%	11	0
h	40%	5	2	14%	36	5
i	33%	15	5	29%	48	14
j	69%	26	18	50%	22	11
k	-	0	0	0%	18	0
end	-	-	-	20%	20	4

Ltgr142	First half			Second half		
Pre note type		n	click		n	click
start	33%	18	6	-	-	-
a	20%	5	1	0%	4	0
b	50%	44	22	64%	56	36
c	20%	15	3	19%	37	7
e	100%	1	1	50%	8	4
f	89%	9	8	52%	23	12
g	8%	13	1	-	0	0
h	19%	58	11	4%	23	1
Post note type						
a	33%	6	2	0%	3	0
b	52%	50	26	56%	50	28
c	74%	19	14	61%	33	20
e	100%	2	2	57%	7	4
f	0%	9	0	0%	23	0
g	0%	13	0	-	0	0
h	14%	64	9	6%	17	1
end	-	-	-	39%	18	7

Ltgr073	First half			Second half		
Pre note type		n	click		n	click
start	95%	20	19	-	-	-
a	0%	62	0	0%	33	0
b	40%	63	25	36%	53	19
c	93%	28	26	83%	36	30
d	0%	6	0	0%	15	0
e	67%	3	2	80%	35	28
Post note type						
a	94%	65	61	93%	30	28
b	0%	68	0	0%	48	0
c	0%	36	0	0%	28	0
d	89%	9	8	100%	12	12
e	75%	4	3	88%	34	30
end	-	-	-	35%	20	7

Ltgr031	First half			Second half		
Pre note type		n	click		n	click
start	70%	20	14	-	-	-
a	47%	47	22	28%	46	13
b	83%	42	35	-	0	0
c	0%	61	0	0%	14	0
d	28%	58	16	21%	43	9
e	21%	29	6	0%	13	0
f	47%	15	7	15%	41	6
g	0%	12	0	0%	26	0
h	43%	7	3	0%	14	0
i	-	0	0	0%	26	0
j	0%	1	0	0%	61	0
Post note type						
a	31%	48	15	13%	45	6
b	100%	42	42	-	0	0
c	47%	62	29	0%	13	0
d	0%	61	0	0%	40	0
e	10%	29	3	8%	13	1
f	32%	19	6	8%	37	3
g	42%	19	8	0%	19	0
h	0%	10	0	0%	11	0
i	-	0	0	4%	26	1
j	0%	2	0	13%	60	8
end	-	-	-	45%	20	9
