# Supplementary material for: The Songbird as a Percussionist: Syntactic Rules for Non-Vocal Sound and Song Production in Java Sparrows
Source: PLoS One. 2015 May 20;10(5):e0124876. doi: 10.1371/journal.pone.0124876 (PMC4438869; doi:10.1371/journal.pone.0124876)
Supplement: S2 Table — (PDF) [file pone.0124876.s006.pdf]

Table S2. Comparisons of bill-click syntax between fathers and their sons. Each line shows the proportion of clicks produced before or after a particular pre- or post-note types. Note names (a–h) are unique to each song family.

Family A	js0002 Father (n = 20 songs)			js0048 Fostered son (n = 20 songs)		
pre note type		n	click		n	click
start	50%	20	10	70%	20	14
a	46%	183	85	4%	116	5
b	1%	72	1	2%	47	1
c	5%	192	9	6%	127	7
d	0%	15	0	0%	1	0
e	93%	14	13	12%	17	2
post note type						
a	39%	183	72	7%	116	8
b	3%	72	2	2%	47	1
c	17%	192	33	16%	127	20
d	0%	15	0	0%	1	0
e	57%	14	8	0%	17	0
end	15%	20	3	0%	20	0

Family B	js0003 Father (n = 20 songs)			js0042 Son (n = 20 songs)			js0044 Fostered son (n = 20 songs)		
pre note type		n	click		n	click		n	click
start	25%	20	5	60%	20	12	15%	146	22
a	38%	108	41	14%	49	7	4%	190	8
b	0%	302	0	1%	126	1	8%	105	8
c	53%	86	46	27%	22	6	14%	35	5
d	10%	106	11	10%	29	3	15%	65	10
e	0%	205	0	0%	100	0	4%	107	4
f	0%	265	0	1%	175	1	18%	60	11
g	1%	142	2	1%	99	1	10%	20	2
post note type									
a	40%	108	43	24%	49	12	18%	146	27
b	19%	302	58	10%	126	13	6%	190	12
c	0%	86	0	0%	22	0	5%	105	5
d	0%	106	0	0%	29	0	0%	35	0
e	0%	205	0	4%	100	4	8%	65	5
f	0%	265	0	0%	175	0	0%	107	0
g	0%	142	0	1%	99	1	22%	60	13
end	20%	20	4	5%	20	1	40%	20	8

Family D	js0016			js0069		
	Father (n = 16 songs)			Son (n = 20 songs)		
pre note type		n	click		n	click
start	0%	16	0	30%	20	6
a	13%	112	15	16%	125	20
b	36%	28	10	29%	65	19
c	2%	47	1	0%	84	0
d	6%	98	6	8%	145	11
e	0%	129	0	1%	185	1
f	0%	24	0	0%	21	0
post note type						
a	6%	112	7	10%	125	13
b	57%	28	16	65%	65	42
c	6%	47	3	2%	84	2
d	1%	98	1	0%	145	0
e	0%	129	0	0%	185	0
f	21%	24	5	0%	21	0
end	0%	16	0	0%	20	0

Family E	js0023			js0051		
	Father (n = 20 songs)			Fostered son (n = 18 songs)		
pre note type		n	click		n	click
start	60%	20	12	0%	18	0
a	26%	35	9	42%	74	31
b	3%	183	5	16%	122	19
c	1%	507	3	2%	374	9
d	3%	90	3	0%	44	0
e	3%	37	1	0%	21	0
f	1%	74	1	5%	37	2
post note type						
a	63%	35	22	42%	74	31
b	2%	183	4	8%	122	10
c	1%	507	7	3%	374	11
d	1%	90	1	5%	44	2
e	0%	37	0	29%	21	6
f	0%	74	0	3%	37	1
end	0%	20	0	0%	18	0

Family G	js0037			js0052		
	Father (n = 18 songs)			Son (n = 20 songs)		
pre note type		n	click		n	click
start	0%	18	0	30%	20	6
a	38%	58	22	86%	7	6
b	6%	155	9	2%	202	5
c	0%	185	0	1%	183	1
d	0%	7	0	7%	15	1
e	16%	95	15	10%	82	8
post note type						
a	28%	58	16	57%	7	4
b	10%	155	16	6%	202	12
c	0%	185	0	0%	183	0
d	0%	7	0	0%	15	0
e	15%	95	14	12%	82	10
end	0%	18	0	5%	20	1

Family B2	js0041			js0087		
	Father (n = 16 songs)			Son (n = 20 songs)		
pre note type		n	click		n	click
start	0%	16	0	65%	20	13
a	10%	114	11	21%	137	29
b	0%	153	0	1%	135	1
c	40%	57	23	52%	61	32
d	13%	52	7	45%	33	15
f	0%	119	0	0%	123	0
g	0%	74	0	0%	66	0
post note type						
a	9%	114	10	26%	137	35
b	20%	153	30	36%	135	48
c	0%	57	0	2%	61	1
d	0%	52	0	0%	33	0
f	1%	119	1	4%	123	5
g	0%	74	0	0%	66	0
end	0%	16	0	5%	20	1
